# Crimean–Congo hemorrhagic fever in the Arab world: A systematic review

**DOI:** 10.3389/fvets.2022.938601

**Published:** 2022-09-13

**Authors:** Nighat Perveen, Gulfaraz Khan

**Affiliations:** ^1^Department of Biology, College of Science, United Arab Emirates University, Al-Ain, United Arab Emirates; ^2^Department of Microbiology & Immunology, College of Medicine and Health Sciences, United Arab Emirates University, Al-Ain, United Arab Emirates

**Keywords:** CCHF, CCHFV, prevalence, distribution, epidemiology, Arab world

## Abstract

Crimean-Congo hemorrhagic fever (CCHF) is an important tick-borne viral infection with a fatality rate of up to 50% during outbreaks. Crimean-Congo hemorrhagic fever virus (CCHFV) is sustained in the ecosystem in benign form through vertical and horizontal transmission cycles involving tick vectors, wildlife, and livestock. Hyalomma ticks are considered the major source of human infection. CCHF occurs most often among butchers, slaughterhouse workers, and farmworkers through infected tick bites or/and contact with blood and tissues of infected livestock. The nosocomial transmission can occur in auxiliary nurses and physicians through contact with the infected patients. The widespread distribution of CCHFV most probably occurred by ticks on migratory birds, or through international travel and trade of livestock and wildlife. During co-infections of ticks and vertebrates, reassortment among genome segments could play a significant role in generating diversity, and hence, a potential risk for the emergence of novel variants. In this systematic review, we aimed to determine the epidemiology, transmission, distribution, mortality, and clinical features of CCHF in 22 Arab countries, comprising the Arab world. Based on the analysis of 57 studies published from 1978 to 2021, we found 20 tick species that could be associated with CCHFV transmission. During the 43-year period, 321 cases of CCHF were reported from 9/22 Arab countries, Iraq, Kuwait, UAE, Saudi Arabia, Oman, Sudan, Egypt, Tunisia, and Mauritania. The mean case fatality rate was 29% during various outbreaks. Individuals working in abattoirs/slaughter houses, livestock farms, and healthcare were most at risk. Contact with blood or body secretions from infected animals and patients was the most common mode of transmission. A number of different animals, including cattle, goats, sheep, and camels were reported to be seropositive for CCHFV. The highest seroprevalence was observed in camels (29%), followed by cattle (21%), goats (15%), and sheep (14%). We discuss these results in the context of policy-making and potential preventative measures that can be implemented to reduce the burden of CCHF in the Arab world.

## Introduction

Emergence or re-emergence of vector-borne zoonotic diseases across the world exhibit the association among pathogens, vectors, animals, and humans, that can lead to health challenges and economic losses (1, 2). Furthermore, vector-borne disease transmission and perseverance mostly rely on overlapping areas/movements of hosts, circulation of competent vectors, and favorable environmental conditions for vector-borne pathogens (3). Ticks are ectoparasites of livestock, wildlife, and humans, and an important vector of viral pathogens. Many tick-borne viral diseases such as Alkhurma hemorrhagic fever (ALKF), Crimean–Congo hemorrhagic fever (CCHF), and Tick-Borne Encephalitis (TBE) have been reported in the Middle East and North Africa (MENA) region (4), where the control of tick vectors continues to be a challenge.

CCHF is a severe tick-borne zoonosis caused by Crimean-Congo hemorrhagic fever virus (CCHFV) (5). It is a biosafety level 4 pathogen with a case fatality rate of up to 50% (6). CCHF has been reported in many countries from Asia, Africa, South-East Europe, and the Middle East (7). In the MENA region, CCHF has been reported from numerous countries (4, 8, 9) and in some of these countries it is endemic (8, 10). Indeed, the incidence of CCHF in the WHO Eastern Mediterranean Region (WHO EMR) appears to have increased in the last decade (8). However, accurate data is lacking, most probably due to the unavailability of comprehensive surveillance systems, and poor understanding of the epidemiology of virus and risk factors of transmission. CCHF is mostly asymptomatic in many animals such as camels, cattle, goats, and sheep (11, 12). Ticks, mainly belonging to the genus *Hyalomma*, act as reservoirs and vectors. Infection in humans occurs through tick bites or by contact with a CCHF infected patient, or by contact with tissues/body fluid or blood of viremic persons and animals (7, 13). CCHF outbreak in the UAE, Oman, and Saudi Arabia with high fatality rate (14, 15) was considered to be associated with the *Hyalomma* tick. Furthermore, human cases were mostly in individuals working in the agriculture and livestock industry (7, 13). Although CCHF/CCHFV has been reported in the Arabian Peninsula (8, 15–25), a detailed and comprehensive picture of the epidemiology, prevalence, mortality rate and clinical features, remains limited. Therefore, we conducted a record-based systematic review and analysis of CCHF in 22 countries of the Arab world from 1978 to 2021 with the aim of filling this gap. We describe the epidemiological characteristics of the disease, provide a record of circulating tick vectors and host species in the region, determine the main routes of transmission of the virus and outline the clinical picture reported in infected cases. Based on our analysis, we suggest potential policies that can be instituted and preventative actions that can be implemented to reduce the burden of CCHF in the region.

## Materials and methods

### Literature search strategy

**Figure 1:**
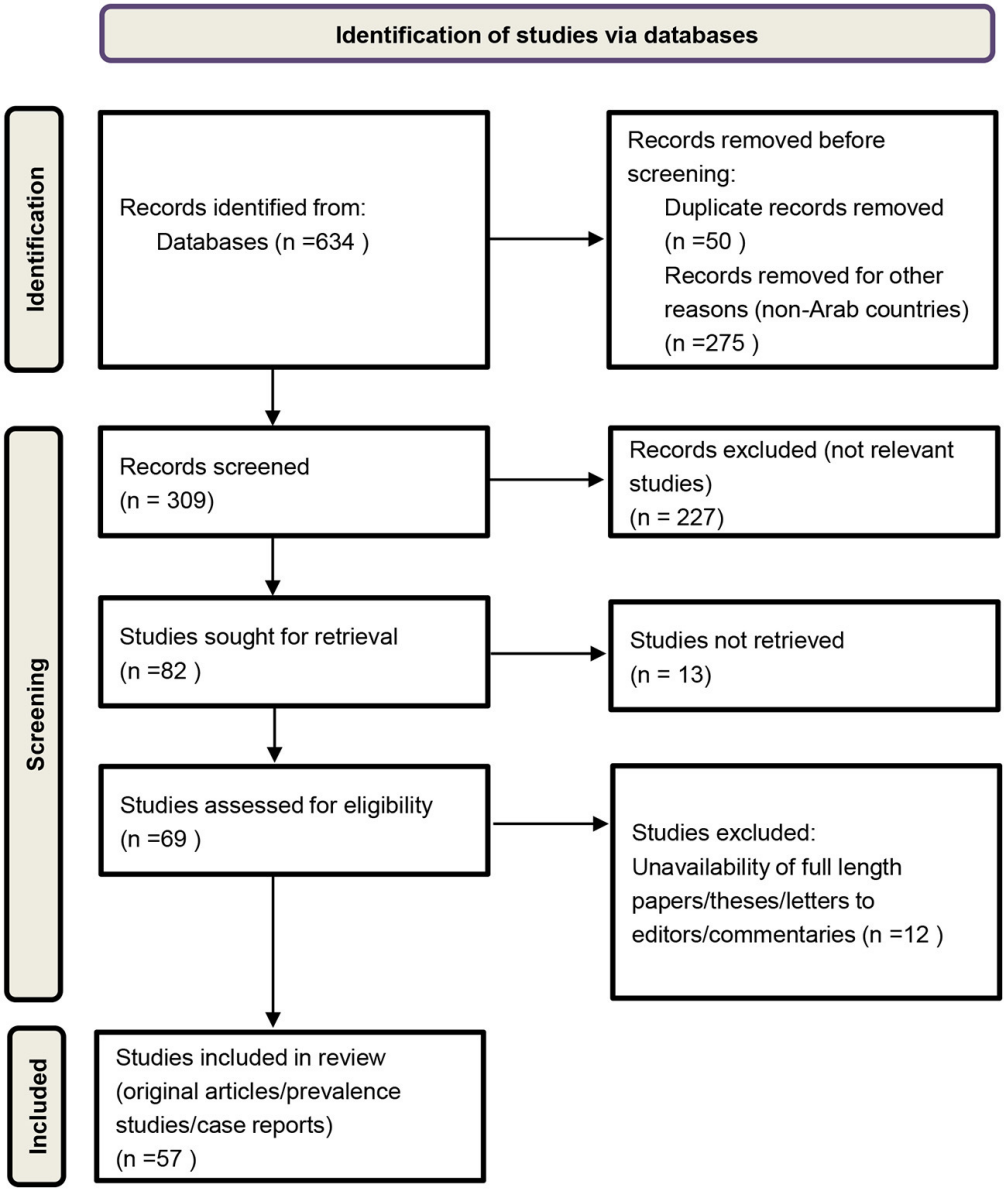
Flow chart diagram for data retrieving and extraction.

In this study, we systemically searched for relevant literature published on CCHF/CCHFV in humans, animals, and ticks in the 22 Arab countries using the Preferred Reporting Items for Systematic Reviews and Meta-Analyses (PRISMA) statement protocol (26) (Figure 1). Our search strategy was based on searching different databases such as Google Scholar, Science Direct, Web of Science, Scopus, and PubMed for retrieving relevant articles published in the Arab countries, from 1978 to 2021. Search terms, “CCHF,” “CCHFV,” “humans” or “patients,” “tick” or “ticks,” “tick vectors,” “animals,” “livestock,” “wildlife,” “small mammals,” and the name of the concerned country were used for retrieving data. The filters were used such as time line (1978–2021) and Arab world/Arab countries.

### Data extraction and quality assessment

The CCHF/CCHFV detection, prevalence, and distribution studies on animals, tick vectors and human case reports with fatality rate were analyzed carefully and reviewed systematically. NP retrieved the data, screened each record/report. GK checked and verified each record/report on the basis of inclusion criteria to avoid duplications and errors to enhance the quality of extracted data. We did not use automation tool. Details are given in PRISMA flow chart diagram (Figure 1). Non-duplicate published records were identified and titles and abstracts were screened. A total of 57 relevant studies specifically on CCHF/CCHFV in Arab countries were identified. For data extraction and qualitative assessment, CCHF case reports, CCHFV screening, prevalence, and distribution studies were analyzed, and duplicates removed. For documentation, we used original research studies and case reports. Letters to the editor and commentaries were excluded. All articles with relevant data according to our searches are summarized in Table 1. Out of all published papers, studies on human cases/clinical reports were more common compared to studies on tick vectors, and serological detection of CCHFV in animals. Some studies were multidimensional and included CCHFV detection from more than one source, for example, ticks, animals, and humans, and these studies were included in data.

**Table 1:** Chronological reporting of CCHFV vectors, hosts, and human cases in the Arab world from 1978–2021.

**Country**	**Vector**	**Host/animal**	**Detection method**	**CCHFV prevalence (%)**	**Year**	**References**
Egypt	*H. anatolicum* *H. marginatum* *H. rufipes* *H. impeltatum* *R. sanguineus* *R. turanicus* *R. annulatus*	Camels, sheep	Serology	Camels: 8.8% Sheep: 23.1%	1978	(17)
Egypt	*H. anatolicum* *H. marginatum* *H. impeltatum* *H. rufipes* *Ha. punctata* *A. variegatum* *H. truncatum* *H. turanicum* *I. ricinus* *A. lepidum* *H. scupense*	Humans, livestock, wild mammals, birds			1979	(27)
United Arab Emirates		Humans	Serology: Immunofluorescence assay (IFA)		1980	(14)
Iraq		Humans	Virus isolation		1980	(28)
Iraq		Humans	Virus isolation		1981	(22)
Iraq		Sheep, goat: Cattle horse, camel small mammals	Serology	Sheep: 57.6% Goat: 49.64% Cattle: 29.28% Horse: 58.73% Camel: 23.23% Small mammals: 14.28%	1981	(29)
United Arab Emirates		Humans	Virus isolation		1981	(30)
Kuwait		Humans	Serology: Immunofluorescence test		1984	(31)
Mauritania		Humans	Serology: Immunofluorescence assay (IFA)		1985	(32)
Egypt		Camel	Serology	Imported camel: 14%	1990	(33)
Mauritania		Humans	Virus isolation		1990	(34)
Mauritania	*H. truncatum*		Virus isolation		1992	(35)
Egypt		Humans	Serology: Enzyme-linked immunosorbent assay (ELISA)	Humans: 1.1%	1994	(36)
Sudan		Humans	Serology (IgM/IgG)		1994	(37)
Oman	*Hyalomma* sp.	Livestock	Serology: ELISA (IgM/IgG)		1996	(15)
United Arab Emirates		Humans	Serology: ELISA (IgM/IgG)		1996	(11)
United Arab Emirates	*H. impeltatum* *H. excavatum* *H. anatolicum*	Camels, cattle, sheep, goats	Serology: ELISA (IgM/IgG)	Livestock market employees: 3% Abattoir employees: 6% Veterinary laboratory: 0% Camel: 7.4% Cattle: 1.7% Sheep: 8.1 % Goat: 12% Ticks: 2.2%	1997	(38)
United Arab Emirates	*Hyalomma* sp.	Livestock	Serology: ELISA RT-PCR	Ticks: 2.19%	1997	(20)
Saudi Arabia		Humans	Serology: IFA ELISA (IgM/IgG)		1997	(39)
Saudi Arabia		Humans, animals	Serology	Humans: 0.8%, Sheep: 4.1%, Goats: 3.2% Cattle: 0.6%. Camel and horse: 0%	1997	(40)
Oman	*H. anatolicum* *R. evertsi*	Domestic livestock	Serology: ELISA (IgG)	Total: 22% domestic animals Cattle: 3% Goats: 27% Sheep: 23% Camels: 16%	2000	(18)
Mauritania	*H. impeltatum* *H. rufipes* *R. evertsi* *H. dromedarii* *R. sanguineus*	Humans Livestock	Serology: ELISA (IgM/IgG) RT-PCR	Total: 17.5% Sheep: 20% Goat: 11% *R. evertsi:* 7%	2004	(41)
Egypt		Cattle, water buffalo sheep, goats	Serology: ELISA (IgG)	Total: 3.13%, Sheep: 6.30% Cattle: 3.83%, Buffalo: 0.38% Goat: 1.14%	2008	(42)
Sudan		Humans	RT-PCR		2010	(43)
Sudan		Humans	Serology: ELISA (IgG) RT-PCR		2011	(44)
Sudan		Humans	Serology: IFA, ELISA (IgM) RT-PCR		2011	(45)
Saudi Arabia		Humans	Serology		2011	(46)
Iraq		Humans	Serology: ELISA (IgM)		2012	(47)
Iraq		Human			2012	(48)
Egypt		Humans			2012	(49)
Egypt		Humans			2012	(50)
Egypt	*H. excavatum* *H. dromedarii*	Livestock	One-Step qRT-PCR	Ticks: 4.34%	2012	(51)
Oman		Humans	RT-PCR		2013	(19)
Sudan		Cattle	Serology: ELISA (IgG)	Cattle: 7%	2013	(52)
Morocco	*H. marginatum*	Migratory birds	Nested PCR	Ticks: 67%	2013	(53)
Egypt		Cow	Serology: ELISA	Cow: 1%	2014	(24)
Iraq		Humans	Serology: ELISA (IgM)		2014	(54)
Sudan		Cattle		Cattle: 19.15%	2015	(25)
Oman	*Hyalomma* sp.	Cattle, camel, sheep, goat	Serology: ELISA (IgG) RT-PCR	Cattle: 17.5% Camel: 15.7% Sheep: 4.3% Goat: 3.8% Ticks: 5.17%	2016	(55)
United Arab Emirates		Humans	Viral PCR testing		2016	(56)
Mauritania		Humans	Serology: ELISA (IgM/IgG) RT-PCR		2016	(57)
Tunisia		Humans	Serology: ELISA (IgM) RT-PCR	Slaughterhouse workers: 5.2% Patients: 2.7%	2016	(16)
Algeria	*H. aegyptium*	Tortoises	Nested reverse transcription PCR	Ticks: 28.6%	2016	(21)
Saudi Arabia	*H. schulzei* *H.onatoli* *H. dromedarii*	Camels and domestic animals	RT-PCR		2017	(58)
Mauritania		Cattle	Serology: ELISA (IgG), IFA (IgG)	Cattle: 67%	2017	(59)
Sudan		Camels	Serology: ELISA (IgM)	Camels: 21.3%	2017	(60)
Oman		Humans	RT-PCR		2019	(61)
Sudan		Humans	Serology: ELISA (IgG)	Patients: 2.6%	2019	(62)
Mauritania	*H. rufipes H. dromedarii H. impeltatum*	Cattle, camels	One-step multiplex real-time RT-qPCR	Total in ticks: 2.56 % *H. rufipes:* 5.67% *H. dromedarii:* 1.89% *H. impeltatum:* 0%	2020	(63)
Egypt		Camels	RT-PCR	Ticks: 1.44%	2020	(64)
United Arab Emirates	*H. dromedarii*	Camels	Serology, Conventional reverse transcription PCRs	Camels: 67%	2020	(10)
Tunisia	*H. impeltatum* *H. excavatum* *H. dromedarii*	Camels	Serology: Enzyme-linked immunosorbent assay (ELISA), RT-qPCR	Camels:89.7% Ticks: 0.61%	2021	(65)
Tunisia	*H. marginatum* *H. impeltatum* *R. sanguineus*	Sheep	Serology: Enzyme-linked immunosorbent assay (ELISA)	Sheep:1.1% Ticks: 0.4%	2021	(66)
Tunisia	*Hyalomma* *Rhipicephalus*	Ruminants	Serology: ELISA (IgG)	Cattle: 11.1% Sheep 6.2 % Goats 7.8%	2021	(67)
United Arab Emirates	*H. dromedarii*	Camels	RT- PCR, Full- length CCHFV genome sequences	Camels: 6.72%	2021	(68)
Egypt	*Hyalomma* sp.	Camels	Nested RT-PCR and Real-time reverse transcription PCR	Ticks: 1.44%	2021	(69)
Mauritania		Livestock	Serology: ELISA (IgG)	Goats and sheep: 15% Cattle: 69% Camels: 81%	2021	(70)

## Results and discussions

**Figure 2:**
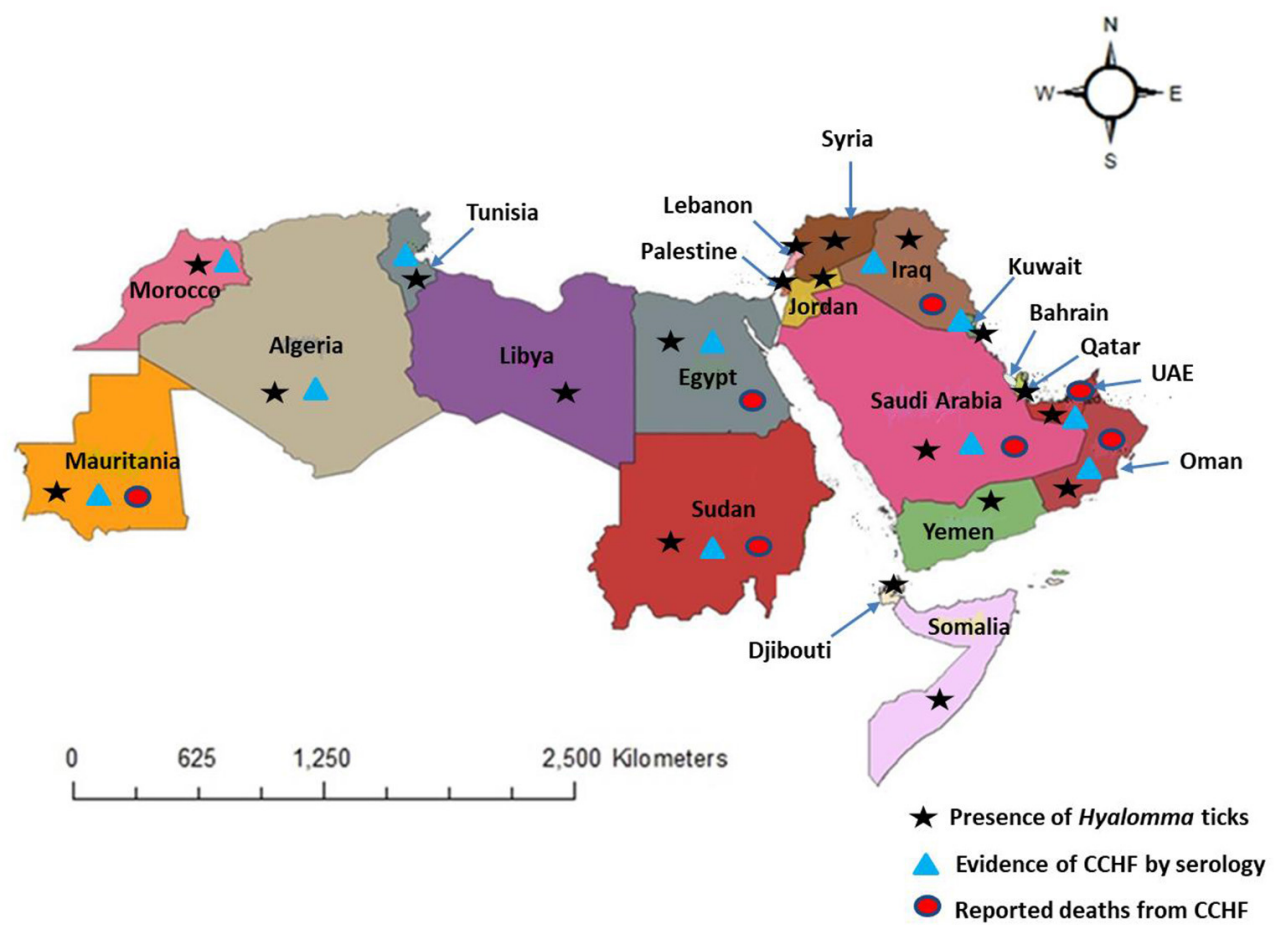
Distribution of *Hyalomma* ticks, evidence of CCHF, and reported deaths from CCHF in the Arab world (map is reproduced published under the Creative Commons Attribution 4.0 International (CC BY 4.0) License in our study, Perveen et al. (4).

**Figure 3:**
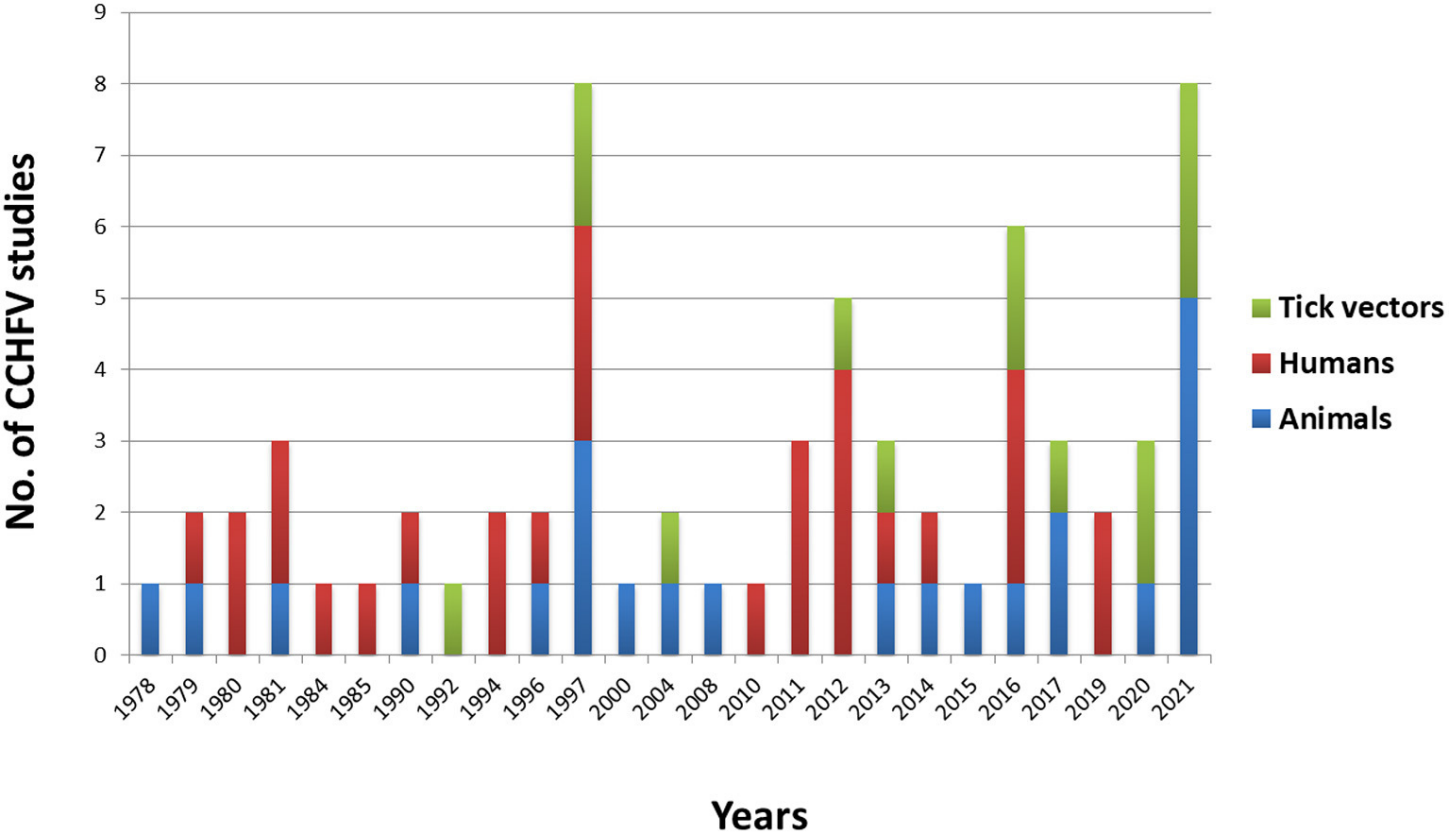
CCHFV reported in ticks, humans and animals in the Arab world from 1978–2021.

In this study, we reviewed literature from 22 Arab countries in the MENA Region, namely Algeria, Bahrain, Comoros, Djibouti, Egypt, Iraq, Jordan, Kuwait, Lebanon, Libya, Mauritania, Morocco, Oman, Palestine, Qatar, Saudi Arabia, Somalia, Sudan, Syria, Tunisia, United Arab Emirates, and Yemen (71) (Figure 2). Approximately 427 million people belong to Arab nations across the world (72). Genus *Hyalomma* is considered the main vector of CCHFV and is found in almost all countries of the region (Figure 2) (4, 73). Anthropogenic changes to the environment in the Arab world, both at small and at large scales, abiotic and biotic factors affect the distribution and abundance of *Hyalomma* ticks and transmission dynamics of the virus. We have documented the presence of tick vectors, serological evidence of CCHFV, its prevalence, and reporting of human cases in different counties of the Arab world from 1978 to 2021 (Table 1). After analysis of 57 studies, CCHFV serological evidence has been recorded from 11 Arab countries including Iraq, Kuwait, UAE, Saudi Arabia, Oman, Sudan, Egypt, Tunisia, Algeria, Mauritania, and Morocco. However, deaths were reported in only seven counties, Mauritania, Oman, UAE, Saudi Arabia, Egypt, Iraq, and Sudan (Figure 2). Fatality rate ranged from 24 to 61% (mean: 29%) during the different outbreaks. Figure 3 illustrates the number of studies published on CCHFV from 1978 to 2021. The mean prevalence of CCHFV antibodies in different hosts and vectors from the published data is given in Table 1. For example, the prevalence of CCHFV antibodies in camels was 29%, in cattle 22%, in buffaloes 0.4%, in sheep 14%, in goats 15%, and in small mammals 14%. Thus, our analysis indicates that camels had the highest seroprevalence of CCHFV in the Arab world. In ticks, the seroprevalence rate was ~10%, as compared to Europe, where CCHFV antibodies were reported as 11.76% in ticks (74).

We established the record of five tick genera, *Amblyomma, Haemaphysalis, Hyalomma, Ixodes*, and *Rhipicephalus*, including 20 species, *Hyalomma aegyptium, Hyalomma anatolicum, Hyalomma excavatum, Hyalomma dromedarii, Hyalomma marginatum, Hyalomma rufipes, Hyalomma impeltatum, Haemaphysalis punctata, Amblyomma variegatum, Hyalomma truncatum, Hyalomma turanicum, Ixodes ricinus, Amblyomma lepidum, Hyalomma scupense, Hyalomma schulzei, Hyalomma onatoli, Rhipicephalus annulatus, Rhipicephalus evertsi, Rhipicephalus sanguineus*, and *Rhipicephalus turanicus* from published data that could be associated with CCHFV transmission (Table 1). Out of the 20 tick species, twelve belong to the genus *Hyalomma*, four belong to the genus *Rhipicephalus* and two belong to the genus *Amblyomma* and one of each belong to *Haemaphysalis*, and *Ixodes*. *Hyalomma* species overlap in the area where other species were found. Focusing on screening of CCHFV, Egypt conducted the greatest number of studies followed by UAE and Sudan (Table 1). However, in some countries of the Arab world, published data on CCHFV was scarce. We found only one published record on CCHFV in Algeria, Kuwait and Morocco. This may be due to a lack of focus of research, low prevalence of the disease in these countries, or poor funding and infrastructure for conducting research. The complex dynamics of host-tick-pathogen system highlights the need for strong interdisciplinary collaborations and teamwork to explain the reasons for recent changes in tick vectors and the virus distribution and abundance. In the following sections, we will discuss CCHFV classification and genome structure, transmission, epidemiology, mortality rate, clinical picture and policy making, relevant to the Arab countries.

### CCHFV classification and genome structure

**Figure 4:**
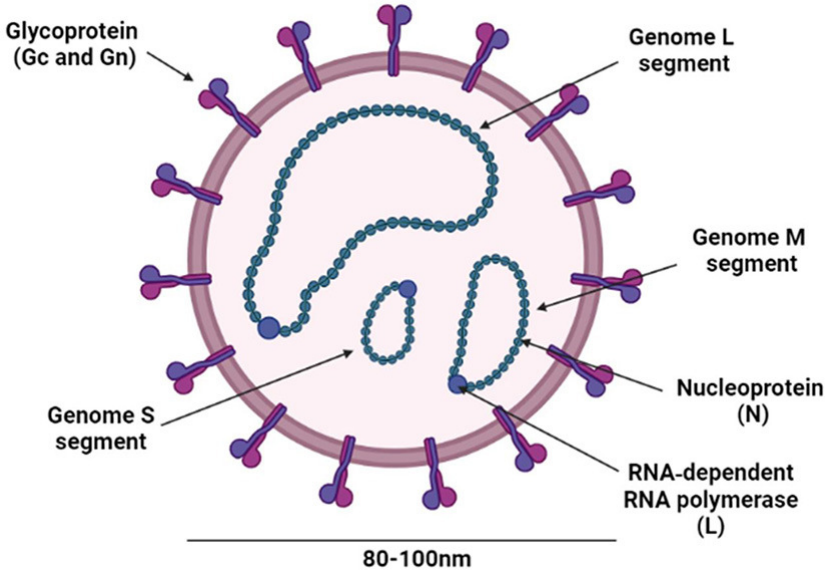
Schematic representation of the CCHFV structure. The figure was created with BioRender (https://Biorender.Com/).

CCHFV is a *Nairovirus* belonging to the family Bunyaviridae that also includes genera *Orthobunyavirus, Hantavirus, Phlebovirus*, and *Tospovirus* (75). All these genera are known to include human pathogens except *Tospovirus* which infects plants (76). Nairoviruses are tick-borne viruses (77, 78) and they are distinguished from other bunyaviruses by their large genome L segments (75, 79). CCHFV is an RNA enveloped virus with a diameter of ~80–100 nm (79) (Figure 4). Its lipid envelope is specked with spikes comprising of the glycoproteins (Gn and Gc), which are responsible for the binding of the virus to cellular receptors. The genome consists of single-stranded RNA with negative polarity, contains three segments, small (S), medium (M), and large (L), encapsidated by the nucleoprotein (NP), and the RNA-dependent RNA polymerase (RdRp), which is required for transcription and genome replication in the host cell (75, 79) (Figure 4).

CCHFV is genetically diverse arbovirus and analysis of complete and partial S segment sequences revealed the seven genetic lineages of CCHFV correlated with the geographical area of virus detection including Asia 1, Asia 2, Africa 1, Africa 2, Africa 3, Europe 1, and Europe 2 (80–82). Reassortment and recombination of segments that occurs with concurrent infections of vectors with viral strains of different lineages may lead to the emergence of new genetic variants of CCHFV (83–85).

Ticks become infected with CCHFV during their blood meal on an infected animal. The virus replicates in the tick midgut, disseminates to the hemocoel, and then spreads to the salivary glands to be transmitted to the next host through saliva. As compared to mosquitoes, ticks feed for a longer period on the host and ingest a greater volume of blood. Ticks digest blood in the acidic intracellular compartments of the gut epithelium (86). Therefore, the virus does not need to bind to a receptor in the tick's midgut to infect and replicate in the cells of the midgut and to spread to different parts of the body such as the salivary glands and reproductive organs (87). The virus passes through several barriers within ticks during the process of replication and transmission. Viral replication is stimulated by the attachment of the tick to the host during the feeding period (6). CCHFV can be associated with the vector for an extended period by persistent infection through the trans-ovarial transmission to the next generation and trans-stadial transmission to the next life stage (6). However, the frequency of both of these transmission processes requires further investigations. For example, ticks can survive long periods without feeding; consequently, tick vectors serve as reservoirs of CCHFV infection even in the absence of vertebrate hosts. For example, in *H. marginatum*, CCHFV was detectable up to 700 days after an infectious blood meal (88). Moreover, ticks have been reported to transmit the virus by biting the vertebrates even after storage at 4 °C for up to 10 months (88).

### Transmission route of CCHFV to humans

**Figure 5:**
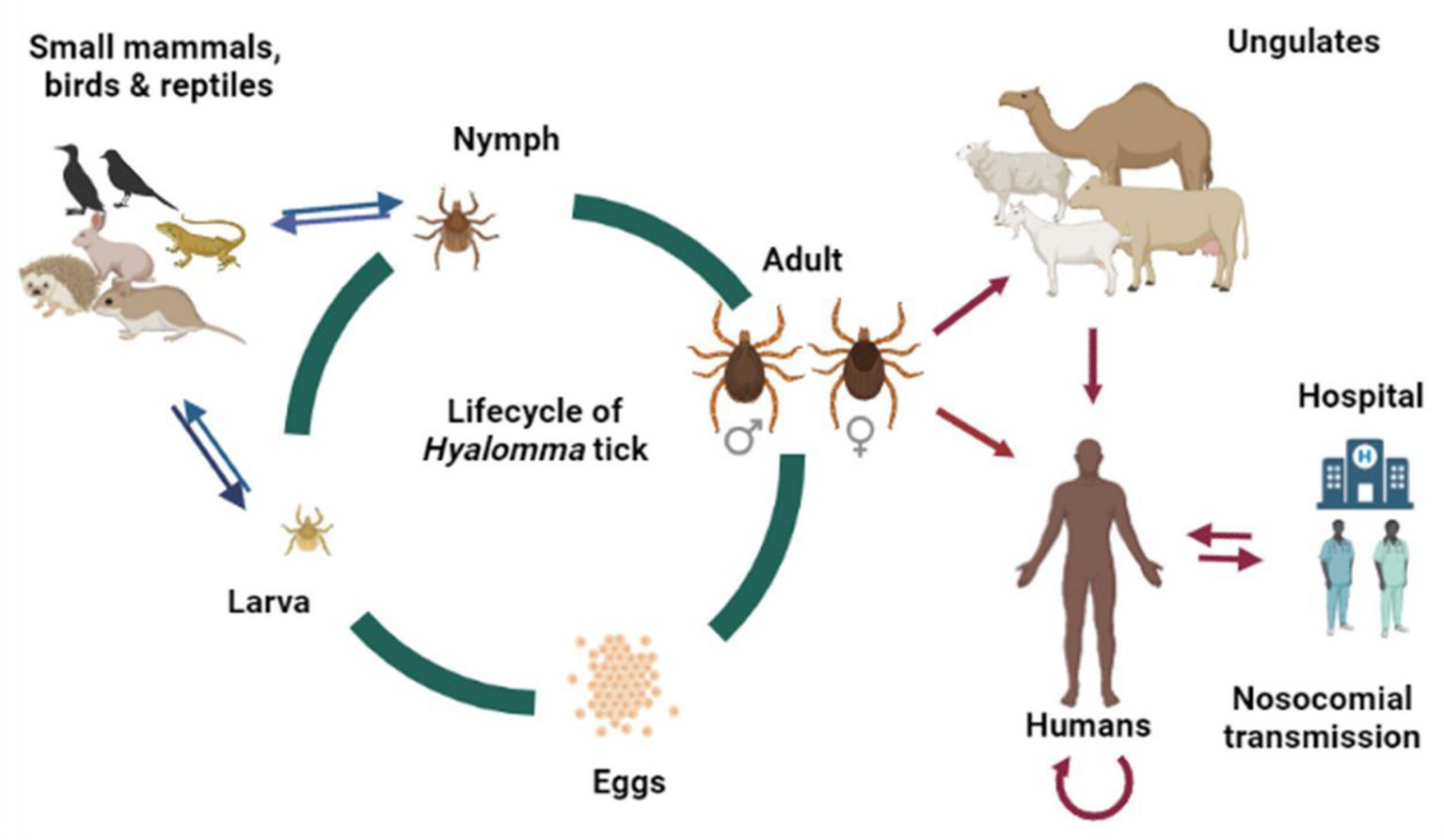
Lifecycle of *Hyalomma* tick and potential transmission route of CCHFV in the Arab world. The original figure was created with BioRender (https://Biorender.Com/).

CCHF infections are enzootic and mostly asymptomatic in various animals (89). The CCHF virus can be transmitted to humans *via* contact with infected humans and animal tissues/blood or by tick bites (90) (Figure 5). Nosocomial outbreaks in hospitals are associated with resource-poor settings (43). For example, a nosocomial outbreak was reported in Al-Fulah, Kordufan, Sudan in 2008 when a 60 years old male patient who had worked as a butcher was admitted to hospital. Due to the lack of personal protective equipment (PPE) and implementation of stringent infection control measures, the virus was transmitted to nurses who had provided care to the index patient (43). The majority of CCHF cases however, have occurred in people associated with the livestock industry, slaughterhouse/abattoir, and veterinary practice (91, 92). In the Arab world, the virus has been shown to circulate in many tick genera (Table 1). However, ticks belonging to the genus *Hyalomma* are the main source of human infections, perhaps due to both immature and adult ticks feeding on host blood that they requires at each stage of their maturation (75). *Hyalomma* ticks act as both reservoirs and vectors for CCHFV (93). The *Hyalomma* tick larvae and nymphs feed on small mammals or/and birds, or reptiles whereas the adults feed on ungulates, and maintain CCHFV in nature through trans-ovarian and trans-stadial transmission (6, 27, 35). The role of reptiles as competent host for CCHFV transmission and as reservoirs needs to be determined. The transmission of CCHFV to animals occurs through a bite of an infected tick. Subsequently, the virus transmits to non-infected ticks while taking blood meal from the infected host. Ticks can also acquire infection during co-feeding of infected and non-infected ticks on same host and viral substances present in the saliva of ticks accelerate the viral transmission (94). However, all mammals are equally susceptible to CCHFV infection (95). Birds are considered poor hosts for CCHFV replication and transmission because birds are commonly resistant to becoming viremic (96). Humans are generally considered as incidental, dead-end host for CCHFV. People predominately get infected through tick bites, contact with tissues and blood of viremic animals, and though tissues and body fluid/blood of infected humans (75). Travel and trade of infected livestock from infected areas to new areas can also lead to CCHFV transmission (97). The threat of CCHFV transmission can be reduced through changes in land use, and by controlling the travel and trade of infected livestock. In the Arab world, during various outbreaks, the most common mode of CCHFV transmission was found to be contact with contaminated blood of carcasses through wounds or mucous membranes of infected animals and patients (Table 1).

### Epidemiology of CCHF in the Arab world

CCHF first caught attention during an outbreak in Crimea in 1944 when 200 Soviet military personnel were infected while assisting farmers (27). It later emerged that the same virus infected a 13-year-old male patient in Congo in 1956, giving the virus its current name (27). It was designated as arbovirus in 1962 (98, 99). CCHF is endemic in Africa, Asia, Eastern Europe, and the Middle East (8, 100). The CCHFV distribution covers the maximum geographic range of any tick-borne virus in the MENA Region. Many Arab countries of the MENA Region have reported CCHF cases. The CCHF geographic distribution overlaps with that of *Hyalomma* ticks (Figure 2). These ticks feed on several animals including livestock and wildlife that could serve as asymptomatic reservoirs of CCHFV in the transmission cycle in endemic areas (89). A wide range of hosts and favorable climatic and ecological conditions in several Arab countries bordering each other could upsurge the incidence of CCHF in the region in the future. Ecological settings and human behavior can also play a crucial role in the maintenance and occurrence of CCHFV within region (101). Furthermore, changes in land-use, urbanization, transportation and trade of infected livestock can also impact the risk of CCHFV transmission. Limitations in surveillance and diagnostic capacities are important impediments in the estimation of the CCHF burden in many countries (102). In the following section we will discuss the epidemiology of CCHFV in specific countries in the Arab world.

#### Algeria

In 2009–2010, a study was conducted in Laghouat Province of Algeria to determine the biological role of *Hyalomma aegyptium* ticks in the epidemiology of CCHF. CCHFV has been detected in *H. aegyptium* ticks collected from tortoises with a prevalence of 28.6% (21). *Hyalomma aegyptium* adults feed mostly on tortoises (103) and less often on hedgehogs and hares. However, larvae and nymphs feed on a wide range of hosts including humans, birds, reptiles, and mammals that increase this species' role as a possible bridge vector for linking wildlife, livestock, and humans to transmit CCHFV (21). No human case has been reported in Algeria (Figure 2).

#### Egypt

Egypt is positioned between numerous foci of CCHFV in Eurasia and Africa. In our data set, Egypt was found to have the highest number of tick species associated with the CCHFV virus (Table 1). Migrating birds during their spring and fall passages (northward- and southward) linked to transport of large numbers of ticks through Egypt from and within the African and Eurasian ranges of CCHFV (17, 27). In 1978, a serological study from Egypt provided the first evidence that antibodies to CCHFV were present in several wild and domestic animals, including camels (8.8%) and sheep (23.1%), and hence, the virus was circulating in country (17). The study also suggested that *H. anatolicum, H. marginatum H. rufipes, H. impeltatum, R. sanguineous, R. turanicus*, and *R. annulatus* were the most common tick species associated with CCHFV. In 2004–2005, to find the role of ruminants as a reservoir host for CCHFV, a serological survey indicated that 3.13% of animals tested were positive for CCHFV antibodies (42). In 1986–1987, 14% of sera of imported camels tested positive for CCHFV antibodies (33). CCHFV antibodies have been reported in other animals including cows (1%) (24), sheep (6.30%) cattle (3.83%), buffaloes (0.38%), and goats (1.14%) (42). Ticks have also been shown to be positive for CCHFV antibodies (51, 64, 69). Human cases, including healthcare workers have also been reported (Table 1). Thus, vector control, continuous screening of domestic animals and strict implementation of infection control measures in healthcare facilities is essential to avoid such outbreaks.

#### Iraq

In 1979, a 24 year-old lady was diagnosed with CCHF at Al-Yarmouk hospital, Baghdad, making her the first confirmed case of CCHF in the country (22). Later two close contacts, one physician and one health worker also contracted the infection and subsequently died (22). Thereafter, CCHF cases were reported in Iraq in different periods from 1980 to 2014 (22, 28, 47, 48, 54) and most of the cases had a history of contact with animals and others were physicians/health workers. Tantawi et al. carried out a study in 1980 to determine the prevalence of CCHFV in animals and most of the animals tested positive with high prevalence for antibodies to the virus (29) (Table 1).

#### Kuwait

From 1979 to 1982, a total of 502 sera samples were collected from two hospitals in Kuwait. Only 18 human cases were found to be positive for CCHFV antibodies (31). Furthermore, *Hyalomma* ticks in Kuwait have been reported in different studies (4, 104), implying that they could be involved in the transmission of CCHFV.

#### Mauritania

In 1983, a 48 year-old male who lived in Selibaby, Mauritania was admitted to a hospital and subsequently confirmed to be positive for CCHFV (32). Five years later, another case was detected (34). In 1992, the sexual and trans-ovarial transmission of CCHFV was determined in *Hyalomma truncatum* ticks, indicating that tick populations could contribute to the maintenance of CCHFV transmission in nature (35). In 2003, there was an urban outbreak of CCHF in which 28.6 % of the cases were fatal (41). Additionally, CCHFV has also been detected in livestock and ticks in different periods (41, 59, 63, 70) (Table 1). In Mauritania, 5–49 cases of CCHF have been reported per year (73).

#### Morocco

In 2013, CCHFV was detected in *H. marginatum* ticks collected from migratory birds in Zouala, Morocco. A total of 546 birds were captured and investigated for ticks. Fifty-two ticks including 19 larvae and 33 nymphs were collected and screened for the virus. Using nested PCR assays (using Eecf primers) 4/6 pools were found positive. All nucleotide sequences showed 100% similarity with the CCHFV strains from Mauritania and Sudan (53). The presence of *H. marginatum* ticks and reservoir of the virus, such as livestock, provide optimal conditions for the establishment of CCHFV in the country. The risk could be increased due to presence of CCHFV in the neighboring country, Mauritania (Figure 2).

#### Oman

A 37 year-old male from Buraimi, Oman, near the UAE border, was diagnosed with CCHF in 1995 (15). On further investigation, this person was staying at the farm having sheep and goats and a few *Hyalomma* ticks were also found on the animals (15). Another case was reported in the same year, but this time in an individual working in a farm in Sur, Muscat, Oman (15). Once again, *Hyalomma* spp. ticks were recovered from livestock on the farm. In 2000, to evaluate the circulation and prevalence of CCHFV in Oman, samples were screened for CCHFV antibodies from workers and animals from different locations, farms, livestock markets, and abattoirs (18). Screening revealed that 30.3% of workers and 22% of animals were positive (18). Tick analysis showed *H. anatolicum* to be the most abundant. In 2011, after 15 years, a 37 years old man was admitted to the Sultan Qaboos University Hospital and diagnosed with CCHFV (19). The patient was started on ribavirin and his condition improved dramatically (19). High prevalence of CCHFV antibodies was reported in cattle (17.5%), camels (15.7 %), goats (4.8%), sheep (4.3%), and ticks (5.1%) (55). From 2011 to 2017, human cases gradually increased and the major risk for CCHFV infections was contact with animals and/or butchering (61). However, no spread in families or healthcare-associated infections were reported (61).

#### Saudi Arabia

CCHF was reported for the first time in the country in 1990 (39) when seven individuals were infected with CCHFV in the city of Mecca. This prompt a study to determine the reason for the outbreak and to understand the epidemiology of CCHF in the region (39). Ticks were collected from livestock and 10/13 ixodid tick species were found to be capable of transmitting the virus. CCHF confirmed and suspected cases had a history of contact with fresh mutton and slaughtering sheep. Twelve fatalities were reported in a serological survey of 40 abattoir workers (confirmed or suspected cases) in Mecca from 1989 to 1990 (39). However, it was suspected that the CCHF virus may have been introduced into the country by infected ticks on imported sheep arriving *via* Jeddah seaport (39, 105). Another survey of CCHFV antibodies was carried out in imported livestock at Jeddah seaport, as well as in humans who had contact with imported animals on farms and in quarantine stations. CCHFV was detected in humans (0.8%), sheep (4.1%), goats (3.2%), and cattle (0.6%) (40), suggesting that the virus was introduced into Saudi Arabia through imported animals. Recently, during an investigation of hemorrhagic fever viruses in the tick populations, *H. schulzei, H. onatoli*, and *H. dromedarii* were found to be positive for CCHFV (58).

#### Sudan

In 1989 an outbreak of acute febrile illness was reported in Northern Sudan coinciding with the presence of phlebotomine sandflies in high density areas. Five human cases tested positive for CCHFV antibodies, along with other viruses (37). During 2008–2009, an outbreak involving seven cases of CCHF was reported in South Sudan (45), indicating both sporadic and nosocomial transmission (43–45). During a seroepidemiological survey to determine the prevalence of CCHFV in North Kordufan State, 7% of cattle tested positive for CCHFV antibodies (52). More recent studies have confirmed the presence of CCHFV in livestock animals, including camels and cattle (25, 60). Recently, human cases of CCHF have been reported from Khashm el Girba, Eastern Sudan (62).

#### Tunisia

Samples from acute febrile patients and slaughterhouse workers were collected in 2014 to investigate the circulation of CCHFV in Tunisia. Ticks were also collected from Northern and Southern Tunisia and examined for the presence of CCHFV. Slaughterhouse workers (5.2%) and patients (2.7%) tested positive with CCHFV antibodies (16). However, no CCHFV infection was detected in ticks. Seroprevalence of CCHFV infection has been reported in one-humped camels (89.7%) in Southern Tunisia (65). Recently, CCHFV infections have also been reported in cattle, sheep, goats, and ticks in different studies (66, 67) (Table 1).

#### United Arab Emirates

A nosocomial CCHF outbreak was reported in Dubai, UAE in 1979 when an index case died just after admission to hospital. Five secondary cases were also identified amongst hospital staff, two of whom died (14). Autopsies on the two fatal cases, confirmed the diagnosis of CCHF (30). Another outbreak of CCHF was reported in UAE during 1994–1995 (11, 38). Investigations revealed CCHFV antibodies in the serum of livestock market employees (3%), abattoir employees (6%), camels (7.4%), cattle (1.7%), sheep (8.1%), goats (12%), and in ticks (2.2%) (38). In 2010, two human cases of CCHF were reported in Dubai (56). More recently, CCHFV antibodies were detected in dromedary camels in two different studies (10, 68) (Table 1). *Hyalomma* ticks are the most prevalent vector species reported on camels, cows, sheep, and goats in the UAE (106–108). Therefore, continued surveillance, monitoring, and screening of tick vectors, animals, and associated people are required to prevent any future CCHF outbreak.

#### Yemen

There is no published record of CCHF infections in humans and animals. However, several tick species have been reported in Yemen that could be potential vectors for CCHFV (27, 109). This creates a huge risk and screening of CCHFV in animal and tick populations is crucial to managing any future infections.

### Clinical picture of CCHF in the Arab world

CCHFV infection can be broadly grouped into four phases: Incubation period, pre-hemorrhagic, hemorrhagic, and convalescent (110). The incubation period is the asymptomatic phase, which persists for 3–7 days after infection. The second is a pre-hemorrhagic phase that lasts for 4–5 days and is characterized by symptoms such as high fever, headache, abdominal pain, myalgia, and hypotension (27). The third phase involves severe symptoms, such as epistaxis, hemoptysis, ecchymosis, diarrhea, neuropsychiatric and cardiovascular changes (7). Severely ill patients can progress to multi-organ failure and death. Those who survive, recovery starts around 10–20 days after the onset of the illness (78). Full recovery can take almost a year in CCHF survivors (13). However, some patients were reported with dramatic recovery in much shorter time (14).

CCHFV causes severe disease in humans with a high fatality rate, up to 50% (111) and up to 80% for nosocomial transmission (112). In Arab countries, mortality rate varied from 24–61% during different outbreaks. Early diagnosis is critical for patient support and for preventing the spread of infection through well-documented human-to-human transmission (113). Ribavirin has been used extensively as an antiviral treatment (113). Table 2 documents CCHF cases and fatality rates reported in different countries in the Arab world over the last four decades.

**Table 2:** Summary of CCHF outbreaks/reports in the Arab world.

**Year**	**Country**	**Confirmed cases**	**Deaths**	**Fatality rate %**	**Reference**
1979-1995	United Arab Emirates	18	11	61	(11, 14)
1979–2014	Iraq	55	24	44	(22, 28, 48, 54)
1979–1982	Kuwait	18	0	0	(31)
1983–2019	Mauritania	50	12	24	(5, 32, 34, 41, 57, 114, 115)
1989–1990	Saudi Arabia	47	12	26	(39)
1995–2017	Oman	88	32	36	(61)
2008–2018	Sudan	34	16	47	(5, 43–45, 116–118)
2014	Tunisia	7	0	0	(16)
**Summary**		321	108	29	

### Policymaking and instituting preventative measures

CCHF is a disease with a high potential of an outbreak with high fatality rate. *Hyalomma* ticks are present across the Arab countries. Climate change and anthropogenic factors could contribute to an extension of the geographic range of CCHFV. Continuous surveillance of tick vectors and animals is required to monitor the CCHF burden and epidemiological trends. Considering the high case fatality rate of CCHF, early detection and diagnosis are critical to allow quick interventions at all levels, including patient, hospital, and community level. Further, the development of a vaccine and new drugs against CCHFV is of major importance. Ribavirin efficacy should be evaluated through well-designed clinical protocols. Awareness about the mode of transmission of CCHF to the general public is essential to curtail the spread in the area. In many rural areas in Arab countries, backyard slaughtering is common practice and this can result in transmission of the virus to humans (8). Similarly, auxiliary staff should be well trained to recognize and act accordingly to avoid nosocomial spread of the infection. High biosafety level laboratories (BLS4) are crucial for rapid confirmation of suspected cases. Tests need to be reliable and affordable. Climate change and anthropogenic factors that may affect the epidemiology of CCHF should be further studied. Risk assessment in CCHF endemic areas is important for devising tick-control strategies. Therefore, multidisciplinary collaboration is required at the local and regional levels to identify relevant gaps and work in an integrated fashion for the prevention and control of CCHF.

### Limitations

This study has a number of limitations. We only included studies/reports published in English. It is possible that some studies may have been published in Arabic which we did not include. Furthermore, we could not find published data on CCHF/CCHFV for some countries. Moreover, reliable and good quality data on CCHF, such as demographics, clinical data, and incidence/fatality rates are not always available or accessible in some of the countries in the region. These limitations can clear impact the analysis.

## Conclusion

CCHF is a zoonotic disease and a public health menace in the Arab world. The geographic range of the disease is mushrooming due to the change in climatic conditions, and travel and trade of livestock. Furthermore, due to alteration in distribution pattern of host range, the distribution of *Hyalomma* ticks is expanding and consequently CCHFV infection risk is increasing. In this systematic review, we have provided a detailed descriptive epidemiology of CCHF in 22 Arab countries. We have discussed the patterns of CCHF at regional as well as country level and suggested strategies which could be implemented to reduce the burden of the disease. Only 9/22 countries, namely, Iraq, Kuwait, UAE, Saudi Arabia, Oman, Sudan, Egypt, Tunisia, and Mauritania have reported cases of CCHF in the literature. Not all countries in the region have the same level of resources or robust surveillance and reporting systems. Thus, the 321 cases of CCHF with 105 deaths reported in the region over a period of 43 years are likely to be underestimates. Outbreaks continue to occur on regular basis. This year in Iraq, there has been an upsurge in the disease to epidemic levels not seen since it was first recorded in 1979; 23 cases and 8 deaths have been reported in just 4 months (https://promedmail.org/ accessed on 24.04.2022). Individuals working in slaughterhouses and veterinarians were found to be most affected in this outbreak. This further highlights the urgent need for establishing effective policies and the strict enforcement of preventative and control measures in countries in the region where they are underdeveloped.

## Data availability statement

The original contributions presented in the study are included in the article, further inquiries can be directed to the corresponding author/s.

## Author contributions

GK checked the record on the basis of inclusion criteria, provided intellectual inputs and shared ideas, conceived, and designed the study. NP searched the literature, screened, and organized the data. NP and GK wrote the manuscript, prepared illustrations, and revised the manuscript. All authors read and approved the final manuscript.

## Conflict of interest

The authors declare that the research was conducted in the absence of any commercial or financial relationships that could be construed as a potential conflict of interest.

## Publisher's note

All claims expressed in this article are solely those of the authors and do not necessarily represent those of their affiliated organizations, or those of the publisher, the editors and the reviewers. Any product that may be evaluated in this article, or claim that may be made by its manufacturer, is not guaranteed or endorsed by the publisher.
